# Finger Vein Recognition Using Local Line Binary Pattern

**DOI:** 10.3390/s111211357

**Published:** 2011-11-30

**Authors:** Bakhtiar Affendi Rosdi, Chai Wuh Shing, Shahrel Azmin Suandi

**Affiliations:** Intelligent Biometric Group, School of Electrical & Electronic Engineering, USM Engineering Campus, Universiti Sains Malaysia, 14300 Nibong Tebal, Seberang Perai Selatan, Pulau Pinang, Malaysia; E-Mails: seng177@gmail.com (C.W.S.); shahrel@eng.usm.my (S.A.S.)

**Keywords:** finger vein, local binary pattern, local line binary pattern, local derivative pattern, biometrics, hand-based biometrics

## Abstract

In this paper, a personal verification method using finger vein is presented. Finger vein can be considered more secured compared to other hands based biometric traits such as fingerprint and palm print because the features are inside the human body. In the proposed method, a new texture descriptor called local line binary pattern (LLBP) is utilized as feature extraction technique. The neighbourhood shape in LLBP is a straight line, unlike in local binary pattern (LBP) which is a square shape. Experimental results show that the proposed method using LLBP has better performance than the previous methods using LBP and local derivative pattern (LDP).

## 1. Introduction

Nowadays, personal verification based on biometric technology has been used in many kinds of applications such as door access control, ATM transactions and border crossing controls. Biometric is the technology of verifying people using human physiological or behavioral features such as fingerprint, iris, face and voice [1]. Due to the fact that a hand contains lots of information and the information is easy to be retrieved, hand based biometrics such as fingerprint [2] and palm print [3] are the most popular biometric technologies.

Fingerprint is the most mature hand based biometric method where it has been used in many applications for years [2]. However, fingerprint based biometric system is vulnerable to forgery because the fingerprints are easily exposed to the others. In addition, the condition of the finger’s surface such as sweat and dryness can prevent a clear fingerprint pattern from being obtained [4]. This can degrade the system’s performance. As for finger knuckle print [5] and palm print [3] based biometric system, it is easy to replicate since the features are external to the human body.

To overcome the limitations of current hand based biometric systems, finger vein recognition had been researched [6]. In [7], they proved that each finger has unique vein patterns so that it can be used in personal verification. Finger vein based biometric system has several benefits when compared with other hands based biometric methods. First, the finger vein pattern is hard to replicate since it is an internal feature. In addition, the quality of the captured vein pattern is not easily influenced by skin conditions. Moreover, as compared with palm vein based verification system [8], the size of the device can be made much smaller. Lastly, finger vein recognition does not require contact between the finger and sensor, which is desirable for a hygienic viewpoint.

Most of the current available approaches for finger vein recognition [6,9,10] have similarities on the feature extraction method which utilized the features from the segmented blood vessel network for recognition. However, due to the optical blurring and skin scattering problems, the finger vein images are not always clear and can show irregular shadings [11]. Therefore, segmentation errors can occur during the feature extraction process due to the low qualities of finger vein images. When the networks are not segmented properly, the recognition accuracy may be degraded.

To solve the problem, [12] proposed a method for finger vein recognition using local binary pattern (LBP) [13] and local derivative pattern (LDP) [14]. In the proposed method, the captured finger vein images are enhanced by modified Gaussian high-pass filter and then LBP and LDP are applied to extract the binary codes from the enhanced images. The similarity between the extracted and enrolled binary codes are measured by Hamming distance. Although the recognition accuracy when LDP is used as feature extraction method is good, the processing time is 2.5 times longer than the LBP. Moreover, the memory size to store the binary codes of LDP is four times bigger than the LBP.

Besides LDP, a numbers of LBP variants have been proposed so far. One of the variants called local ternary pattern (LTP) has been proposed in [15]. LTP uses three-value encoding instead of two-value encoding as in the original version of LBP. Another variant of LBP that uses five-value encoding called local quinary pattern (LQP) has been proposed in [16]. Recently, [17] proposed a new variant of LBP called local line binary pattern (LLBP) and applied it to face recognition. They demonstrated that the proposed method can produce higher recognition rates compared to LBP on two benchmark face databases.

**Figure 1.**
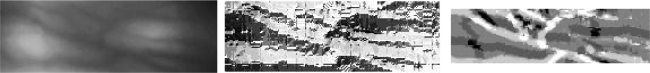
The finger vein image (left), the image after processed by LBP (middle) and the image after processed by LLBP (right).

The problems of the LDP and the advantages of LLBP have motivated us to use LLBP as feature extraction technique in finger vein recognition. The main difference between LLBP and LBP/LDP is its neighbourhood shape is a straight line with length *N* pixel, unlike in LBP/LDP, which is a square. We believe that the straight-line shape of LLBP is more suitable to capture the pattern inside a finger vein image. The finger vein pattern is clearer in the image processed by LLBP than the image processed by LBP as shown in Figure 1. It should be noted that for viewing convenience, the binary codes computed for each pixel in Figure 1 (left) using LBP and LLBP are converted to decimal numbers, which are then normalized to values ranging within 0 to 255 to represent the grayscale values. The resulting images are illustrated in Figure 1 (middle) and Figure 1 (right) for LBP and LLBP, respectively.

## 2. Proposed Method

**Figure 2.**
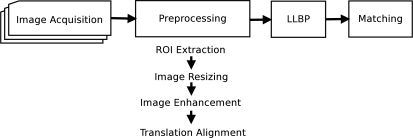
Block diagram of the proposed method.

Figure 2 shows the block diagram of the proposed method for finger vein recognition. The method consists of four main stages: image acquisition, preprocessing, feature extraction by Local Line Binary Pattern (LLBP) and the calculation of matching scores by Hamming distance.

### 2.1. Finger Vein Image Acquisition

**Figure 3.**
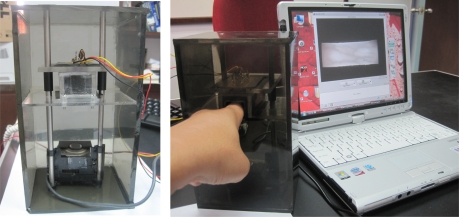
Finger vein image capturing device.

To capture the finger vein images, a special imaging device is constructed as shown in Figure 3 [18]. The constructed device consists of a modified camera (Logitech V-UAV35) and an array of infra-red LED (OSRAM SFH485, wavelength = 880 nm). The camera is not infra-red (IR) sensitive device where it consists of an IR blocking filter. Hence, the IR blocking filter is replaced by a negative film to react as an IR pass filter. To reduce the finger alignment problem, especially finger rotation, an open window with a fixed size (2.5 cm × 2.5 cm) is set for the user to place their finger during the capturing process.

### 2.2. Preprocessing

There are four major steps in the preprocessing stage, which are ROI extraction, image resizing, image enhancement and translation alignment.

**Figure 4.**
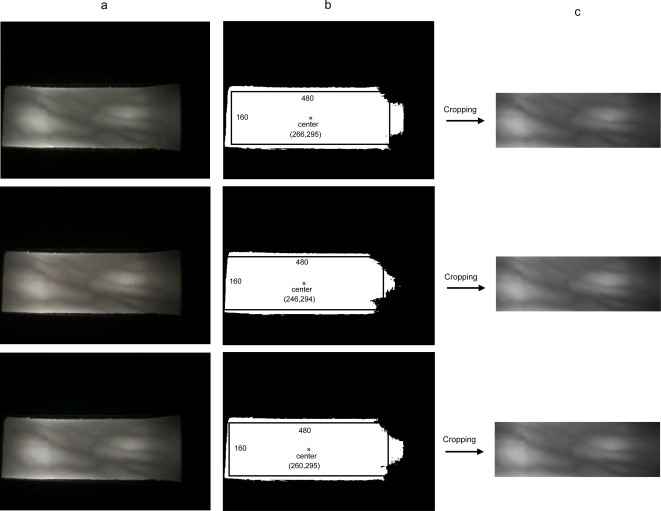
Example of **(a)** the captured images, **(b)** the binarized images with the center of the objects and **(c)** the cropped images for a finger at intervals.

The original image is acquired with the black unwanted background. A simple algorithm is developed to extract the finger vein image from the background. Three major steps involved in this algorithm. First, the captured image is binarized [19] using a threshold value that has been determined by Otsu’s method [20]. Then, the center of the object, which is the finger, is obtained [21]. Finally, the image is cropped to 480 × 160 pixels based on the obtained center of the finger. Figure 4 (a–c) shows the captured, the binarized, and the cropped images for a finger at intervals, respectively. As shown in Figure 4 (b), the center of the objects for a same finger captured at an interval are different. This is because, our device only can reduce the finger rotation problem and not the horizontal and vertical displacement problems.

To reduce the time complexity and eliminate pixel noise, the cropped image is resized into smaller size. The cropped image size of 480 × 160 pixels is reduced to a resolution, 192 × 64 pixels, in which the resize ratio is 0.4.

In general, the finger vein image is in low contrast due to the variation of finger profile. As in [12], a symmetrical modified Gaussian high-pass filter is used to enhance the contrast of the finger vein image. The filter has the following formula :

(1) $$H(x,y)=a\left(1-e^{-D^{2}(x,y)/2D_{0}^{2}}\right)+b$$

**Figure 5.**
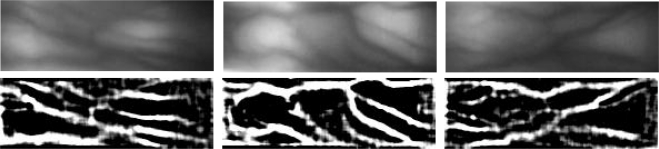
The resized (top) and their enhanced images.

Here, *D*(*x, y*) is defined as follows:

(2) $$D(x,y)={\left[{\left(x-x_{0}\right)}^{2}+{\left(y-y_{0}\right)}^{2}\right]}^{1/2}$$

where *D*(*x, y*) is the distance between the center and a relative position, *x* and *y* are the positions relative to the center *D*_0_(*x*_0_*, y*_0_) of a convolution mask, and *a* and *b* are adjustment variables that can change the amplitude and DC level of the filtering mask. In this paper, an experiment is designed to determine the optimum size of the filtering mask and the values of adjustment variables *a* and *b* for this particular finger vein recognition. Examples of finger vein images and their convolved results are shown in Figure 5. In these examples, the size of the filtering mask is 9 and, the variables *a* and *b* are 12.53 and −4, respectively.

Although our proposed ROI extraction algorithm can reduce the horizontal and vertical displacement between the extracted images, the alignment problems still cannot be totally eliminated. In contrast to [12] and [22], these displacement problems are solved by evaluating the translation parameters between two enhanced images using the phase only correlation (POC) function [4,5]. Then, the common regions for the two images are cropped based on the evaluated translation parameters. The translation parameters (*t_x_*, *t_y_*) between two enhanced images *f* and *g* can be estimated from the peak location of the POC function of them. Then, *f* and *g* are aligned based on (*t_x_*, *t_y_*) and the common regions *f_c_* and *g_c_* are extracted. It should be noted that, the two enhanced images only will be aligned when *t_x_* and *t_y_* are less than 20 and 10, respectively. Generally, when *t_x_* and *t_y_* are larger than those values, the two images are most likely from two different fingers.

### 2.3. Feature Extraction

In [12], LDP and LBP are used to extract the binary codes from the enhanced images. Although the performance of LDP is better than the LBP, the computation time for LDP is about 2.5 times slower than the LBP. Moreover, the code length for LDP is four times longer than the LBP. The computation time and template size are two important factors that need to be considered in designing a biometric system. To overcome the above-mentioned problems, the binary codes in this work are extracted from the enhanced images using a new texture descriptor called Local Line Binary Pattern (LLBP) [17]. One of the benefits of LLBP operator is that it can emphasize the change in image intensity such as vertices, edges and corners.

**Figure 6.**
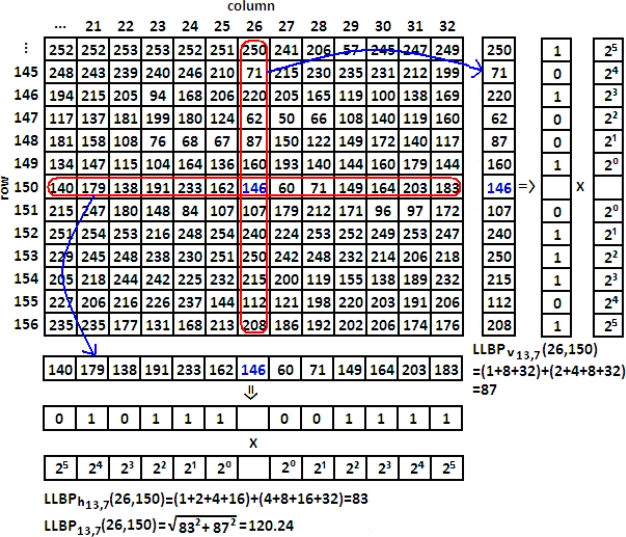
Example of LLBP operator.

Motivated by LBP, Petpon and Srisuk [17] proposed an LLBP operator for face recognition. The operator consists of two components: horizontal component and vertical component. The magnitude of LLBP can be obtained by calculating the line binary codes for both components. The illustration of LLBP operator is shown in Figure 6, and its mathematic definitions are given in Equations (4)–(6). *LLBP_h_*, *LLBP_v_* and *LLBP_m_* are LLBP on horizontal direction, vertical direction, and its magnitude, respectively. *N* is the length of the line in pixel, *h_n_* is the pixel along with the horizontal line and *v_n_* is the pixel along with the vertical line, $c=\frac{N}{2}$ is the position of the center pixel *h_c_* on the horizontal line and *v_c_* on the vertical line, and *s*(·) function defines a thresholding function as in Equation (3).

Employing Equations (3) and (4), the horizontal component of LLBP (*LLBP_h_*) extracts a binary code of *N* − 1 bits for each pixel. The same numbers of bits are extracted by the vertical component of LLBP (*LLBP_v_*) using Equations (3) and (5). Consequently, by concatenating the binary codes from *LLBP_h_* and *LLBP_v_*, the total binary code of LLBP for each pixel is 2(*N* − 1) bits. In Figure 6, the binary sequence for horizontal (vertical) component is defined from left (top) as 010111001111_(2)_(101001011101_(2)_). Hence, the binary code for LLBP is 010111001111101001011101_(2)_.

(3) $$s(x)=\{\begin{array}{ll} 1, & x\geq 0,\\ 0, & x<0. \end{array}$$

(4) $$\begin{array}{ll} {\mathrm{LLBP}}_{\mathrm{hN},c}(x,y) & =\sum_{n=1}^{c-1}s\left(h_{n}-h_{c}\right)\cdot 2^{c-n-1}\\ & +\sum_{n=c+1}^{N}s\left(h_{n}-h_{c}\right)\cdot 2^{n-c-1} \end{array}$$

(5) $$\begin{array}{ll} {\mathrm{LLBP}}_{\mathrm{vN},c}(x,y) & =\sum_{n=1}^{c-1}s\left(v_{n}-v_{c}\right)\cdot 2^{c-n-1}\\ & +\sum_{n=c+1}^{N}s\left(v_{n}-v_{c}\right)\cdot 2^{n-c-1} \end{array}$$

(6) $$\mathrm{LLB}P_{m}=\sqrt{\mathrm{LLB}P_{h}^{2}+\mathrm{LLB}P_{v}^{2}}$$

### 2.4. Matching

As in [12], the similarity between the extracted binary codes and the enrolled codes is measured using Hamming Distance (HD). The formula is given in Equation (7).

(7) $$\mathrm{HD}=\frac{\left\|\left(\mathrm{codeA}⊗\mathrm{codeB}\right)\right\|}{\mathrm{CodeLength}}$$

where ⊗ is a Boolean exclusive-OR operator between corresponding pair of bits. The *codeA* and *codeB* are the extracted binary and enrolled codes, respectively. *CodeLength* is the total number of bits of the enrolled codes. The HD value is ranging from 0 to 1. The HD is close to 0, when the two codes are from the same finger. When the two codes are from two different fingers, the HD is close to 1.

## 3. Experimental Results

Two major experiments have been conducted using our own established database [18]. First, an experiment to determine the optimum size of the filtering mask for modified Gaussian high-pass filter and the optimum length of the line *N* for the LLBP and *LLBP_v_* is conducted. Then, the performance of the LLBP and *LLBP_v_* operators in personal verification are compared with the previously proposed methods [12] which utilize LBP and LDP. The number of sampling points and radius for the LBP that has been used in [12] are 8 and 1, respectively (denoted as LBP (8, 1)). In this paper, the performance of our proposed method is also compared to LBP with the number of sampling points and radius are, 8 and 2, respectively (denoted as LBP (8, 2)). Moreover, we compare the performance of our method with another two variants of LBP, which are LDiP [23] and LTP [15] as well. The performance is evaluated based on Equal Error Rate (EER) as in [10,12,22]. The EER is defined as the error rate when the False Acceptance Rate (FAR) and the False Rejection Rate (FRR) are equal. FAR is the error rate where the un-enrolled finger vein images are accepted as enrolled images. FRR is the error rate where the enrolled finger vein images are rejected as un-enrolled images.

### 3.1. Database of the Finger Vein Images

**Figure 7.**
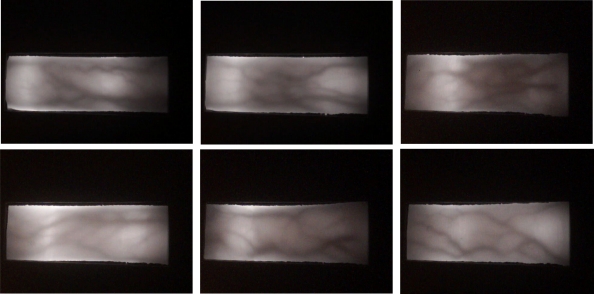
Examples of the captured finger vein images.

The finger vein images that have been used in this study were collected using the capturing device that was explained in Section 2.1. The images were collected from 51 male and female volunteers, who are staffs and students of Universiti Sains Malaysia. The age of the subjects ranged from 21 to 56 years old. Each subject provides 10 images of four fingers, which are left index, left middle, right index and right middle fingers. Consequently, there are 51 sets of four fingers with 10 images for each finger. In total, the database contains 2040 images from 204 different fingers. The spatial and depth resolutions of the captured finger vein images were 640 × 480 and 256 gray levels, respectively. Figure 7 shows examples of finger vein images captured using our device.

### 3.2. Determination of Parameters

There are two parameters that will affect the verification accuracy of the proposed method: the size of the filtering mask (*S*) for modified Gaussian high-pass filter and the length of the line (*N*) for the LLBP and *LLBP_v_* operators. In order to determine the optimum values for these two parameters, an experiment was conducted. A sub-dataset which contained 200 finger vein images from 20 different fingers were used in the experiment. The tuning criterion was the parameters that could lead to a lower EER would be chosen. For a fair comparison, the optimum size of the filtering mask for the LBP (8, 1), LBP (8, 2), LDP, LDiP and LTP have also been determined in the same manner.

**Figure 8.**
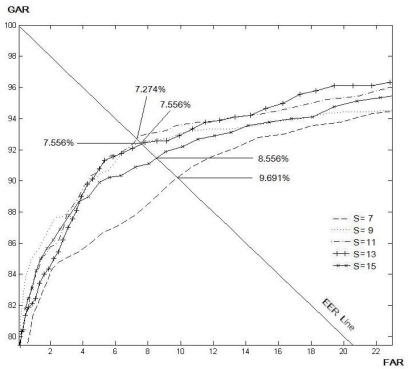
EERs (%) by varying *S* for LBP (8, 1) based on a sub-dataset of finger vein images.

**Figure 9.**
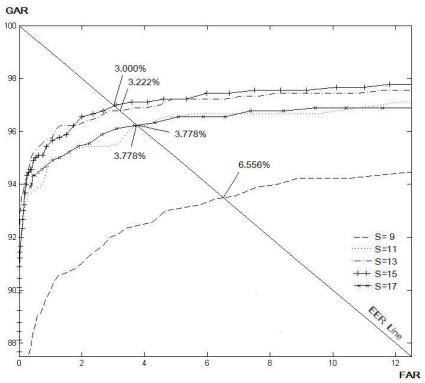
EERs (%) by varying *S* for LBP (8, 2) based on a sub-dataset of finger vein images.

**Figure 10.**
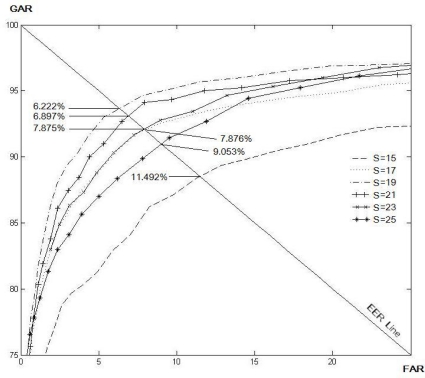
EERs (%) by varying *S* for LDP based on a sub-dataset of finger vein images.

**Figure 11.**
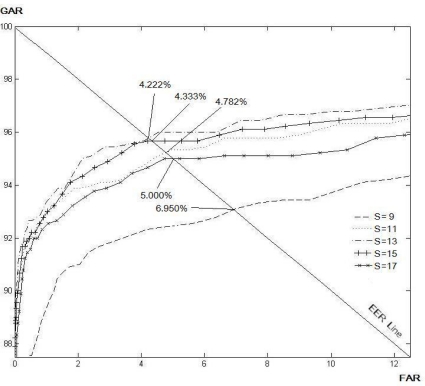
EERs (%) by varying *S* for LDiP based on a sub-dataset of finger vein images.

**Figure 12.**
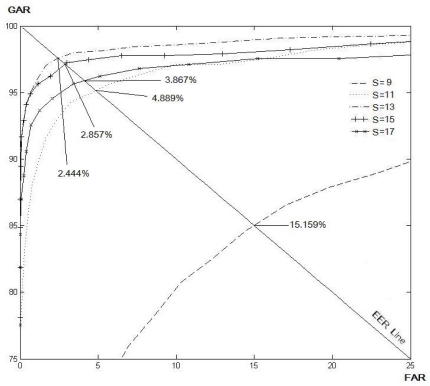
EERs (%) by varying *S* for LTP based on a sub-dataset of finger vein images.

Tables 1 and 2 show the EER for the various size of *N* and *S* for the LLBP and *LLBP_v_* operators, respectively. As a result, the optimal matching performance for the LLBP is observed when the length of the line (*N*) is 21, and the size of the filtering mask (*S*) is 15. As for the *LLBP_v_*, the optimum values of *N* and *S* are 17 and 15, respectively. Figures 8–12 show the EER for the various size of *S* for the LBP (8, 1), LBP (8, 2), LDP, LDiP and LTP operators, respectively. The optimum size of the filtering mask for LBP (8, 1), LBP (8, 2), LDP, LDiP and LTP are 11, 15, 19, 13 and 13, respectively.

### 3.3. Verification Results

**Figure 13.**
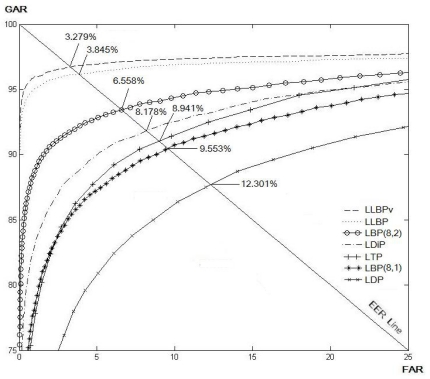
EERs (%) according to various operators based on the whole dataset of finger vein images.

**Figure 14.**
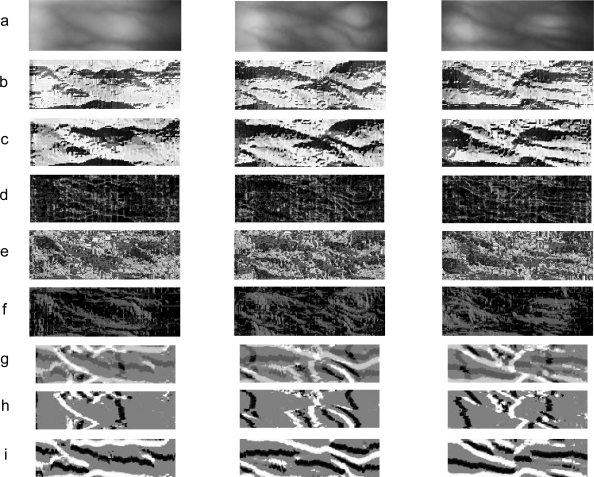
Example of **(a)** the cropped images and the images after processed by various texture descriptors (**(b)** LBP (8, 1), **(c)** LBP (8, 2), **(d)** LDP, **(e)** LDiP, **(f)** LTP, **(g)** LLBP, **(h)** *LLBP_h_*, and **(i)** *LLBP_v_*) for three different fingers.

In order to show the superiority of the local line binary pattern in personal verification, the proposed method was compared with the other variants of LBP [12,15,23]. In the experiment, the optimal parameter values obtained in previous experiment are employed. The experiment was conducted on all finger vein images. Thus, there were 204 fingers (51 × 4) and 10 images for each finger. As a result, the number of genuine tests is 9180 $\left(204\times \left(\begin{array}{c} 10\\ 2 \end{array}\right)\right)$ and the number of imposter tests is 2,070,600 $\left(\left(\begin{array}{c} 2040\\ 2 \end{array}\right)-9180\right)$. The results in terms of EER are summarized in Figure 13. From the results shown in Figure 13, it is observed that the performance of the proposed method performs significantly better than the other LBP variants. The verification accuracy for *LLBP_v_* is slightly better than the LLBP. As shown in Figure 14, the vein is much clearer in the image processed by *LLBP_v_* compared with the image processed by the other LBP variants. We believe that the vertical component of the local line binary pattern can extract significant and important features by emphasizing the vein line. It should be noted that for viewing convenience, the binary codes computed for each pixel in Figure 14 (a) using LBP variants are converted to decimal numbers, which are then normalized to values ranging within 0 to 255 to represent the grayscale values. The resulting images are illustrated in Figure 14 (b–i), for LBP (8,1), LBP (8,2), LDP, LDiP, LTP, LLBP, *LLBP_h_*, and *LLBP_v_*, respectively.

### 3.4. Speed and Memory

All experiments were performed using MATLAB on an Intel Core i5 processor and 4 GB RAM. The processing time and binary codes length of the proposed method were compared with the other LBP variants. From the results shown in Table 3, it is obvious that the processing time for the *LLBP_v_* is significantly faster than the other LBP variants. As for the binary code length, *LLBP_v_* produces much shorter length than the previously proposed method using LDP.

## 4. Conclusions

Extraction of robust features from finger vein images is an important issue in a finger vein based biometric system. Instead of using LBP and LDP, we propose to use the local line binary pattern (LLBP) as feature extraction technique. The straight-line shape of LLBP can extract robust features from the images with unclear veins. Experimental results on the images from 204 fingers that are captured from our own prototype device indicate that the equal error rate (EER) for the LLBP is significantly lower than the LBP and LDP. Moreover, the feature extraction time for LLBP is faster than the other LBP variants. We also find out that the vertical component of LLBP is the most suitable for finger vein recognition. In future, we plan to fuse the features from the finger vein images with the shape of a finger using various fusion techniques. We hope that the EER can be further reduced by the fusion of these two features.

## Figures and Tables

**Table 1.** EERs (%) by varying *N* and *S* for LLBP based on a sub-dataset of finger vein images (N: length of the line, S: size of the filtering mask.)

*S*	11	13	15	17	19
*N*					

17	3.33	2.11	2.11	2.78	2.67
19	3.22	2.11	2.11	2.56	2.56
21	3.22	2.11	**1.89**	2.56	2.56
23	3.22	2.11	1.89	2.56	2.33
25	3.22	2.00	1.89	2.44	2.33

**Table 2.** EERs (%) by varying *N* and *S* for *LLBP_v_* based on a sub-dataset of finger vein images (N: length of the line, S: size of the filtering mask.)

*S*	11	13	15	17	19
*N*					

13	3.11	2.33	1.89	2.44	2.33
15	3.11	2.03	1.89	2.33	2.22
17	3.00	2.00	**1.78**	2.33	2.22
19	3.00	2.00	1.78	2.22	2.17
21	2.89	2.00	1.78	2.22	2.12

**Table 3.** Comparison of processing time and binary code length.

Operator	Feature extraction (ms)	Matching (ms)	Total processing time (ms)	Code Length (bits)

*LLBP_v_* (N = 17)	29.6	7.9	37.5	135,168 (176 × 48 × 16)
LLBP (N = 21)	59	8.1	67.1	302,720 (172 × 44 × 40)
LBP (8, 1) [12]	65.1	10.3	75.4	94,240 (190 × 62 × 8)
LBP (8, 2)	439.2	10.3	449.5	90,240 (188 × 60 × 8)
LDP [12]	193.1	11.7	204.8	360,960 (188 × 60 × 32)
LDiP [23]	713.3	10.3	723.6	94,240 (190 × 62 × 8)
LTP [15]	82.6	11	93.6	188,480 (190 × 62 × 16)
